# Enhancement and Segmentation Workflow for the Developing Zebrafish Vasculature †

**DOI:** 10.3390/jimaging5010014

**Published:** 2019-01-11

**Authors:** Elisabeth Kugler, Karen Plant, Timothy Chico, Paul Armitage

**Affiliations:** 1 Department of Infection, Immunity and Cardiovascular Disease, Faculty of Medicine, University of Sheffield, Sheffield S10 2JF, UK; 2 The Bateson Centre, Firth Court, University of Sheffield, Western Bank, Sheffield S10 2TN, UK

**Keywords:** 3D, analysis, development, *in vivo*, light sheet fluorescence microscopy (LSFM), segmentation, vasculature, zebrafish

## Abstract

Zebrafish have become an established *in vivo* vertebrate model to study cardiovascular development and disease. However, most published studies of the zebrafish vascular architecture rely on subjective visual assessment, rather than objective quantification. In this paper, we used state-of-the-art light sheet fluorescence microscopy to visualize the vasculature in transgenic fluorescent reporter zebrafish. Analysis of image quality, vascular enhancement methods, and segmentation approaches were performed in the framework of the open-source software Fiji to allow dissemination and reproducibility. Here, we build on a previously developed image processing pipeline; evaluate its applicability to a wider range of data; apply and evaluate an alternative vascular enhancement method; and, finally, suggest a work-flow for successful segmentation of the embryonic zebrafish vasculature.

## 1. Introduction

Zebrafish are an increasingly used vertebrate model to study cardiovascular development and disease. Characteristics such as larval transparency, high progeny rates, and *ex utero* development make them an excellent model to study vascular architecture *in vivo* [1]. Transgenic fluorescent reporter lines make it possible to visualize certain cell types of the vasculature non-invasively [2]. Such transgenics have largely replaced the need for laborious micro-angiography to visualize vessels; meaning fish can be imaged more efficiently, allowing higher replicability and sample numbers. Moreover, due to the emergence of new imaging modalities with increased penetration depth and reduced photo-toxicity, such as light sheet fluorescence microscopy (LSFM), it is possible to study the vasculature even in deep anatomical layers for durations of days rather than minutes or hours [3,4].

Quantification of the vascular architecture (e.g., vessel volume, diameter, length, branching points, etc.) from imaging modalities such as CT and MRI is commonly used in the medical field, providing a read-out that describes the physiological health status of the vasculature [5]. Such quantitative approaches are less extensively adopted in pre-clinical models, with the vascular phenotype often only being assessed by subjective assessment. This lack of objective 3D quantification is especially pronounced for the zebrafish cranial vasculature and, also, for data acquired with fluorescence microscopy in transgenic samples. A few previous studies have adopted quantitative approaches to investigate the zebrafish vasculature, the majority of which focused on the trunk vasculature in images acquired after micro-angiography [6,7,8,9], while another trunk vasculature segmentation approach was briefly reported for transgenic fish [10]. Segmentation and quantification of sub-regions of the cranial vasculature has previously been performed in [11] and [12], but both these studies utilized commercial software and lacked sufficient documentation for replicability.

A possible reason for the shortage of zebrafish vasculature 3D quantification methods is that transgenic reporters for endothelial cell visualization outline the vascular lumen, resulting in a cross-vessel double-peak intensity distribution of lumenised vessels [13], while non-lumenised and small vessels are visualized with a single-peak distribution. This contrasts with clinical imaging modalities (e.g., CT and MRI) for which most vascular quantification methods have been developed, as these tend to image blood within the vessels, providing a single-peak intensity distribution across the vessel profile. Most existing quantification methods have been optimised for this single-peak distribution and are not directly transferable to images of transgenic zebrafish. Also, data acquisition with fluorescence microscopy often leads to a higher data load (e.g., LSFM 0.3 × 0.3 × 0.5 µm; approx. 500 slices) compared to images acquired with CT or MRI (e.g., MRI 1 × 1 × 1 mm; approx. 100 slices). This can further hinder the direct translation of methods developed for medical imaging, as run-time and memory issues can arise when applying these to the larger datasets typically generated by LSFM. Hence, image analysis methods for the zebrafish vasculature not only need to consider double- and single-peak intensity distributions across vessels, but also be able to handle large datasets computationally.

To understand image properties prior to analysis, we previously studied image quality in different transgenic reporter lines using vascular contrast-to-noise ratio (CNR) as a quantitative indicator. This enabled us to obtain a quantitative metric of image quality for each of the studied transgenic lines [13]. Our findings indicated that, while there was some variability in CNR between transgenic lines, CNR remained sufficiently high in all examined lines that reliable segmentation should be feasible.

Nevertheless, there was enough variability to suggest that CNR should be considered when developing and evaluating different segmentation approaches. In this work, we perform further examination to improve the robustness of our previous findings. In addition, we investigate the extent to which CNR levels vary during early embryonic development to analyse whether developmental differences in image quality should be considered. Furthermore, we previously quantified CNR levels in vessels of different size to analyse whether CNR levels depend on vessel diameter and found that there was no significant difference in CNR between the different vessels. In this work, we include data from an additional experimental repeat; again, to improve robustness of the previously found results.

The requirement for and ability to correct sample motion was previously assessed in the transgenic *Tg(kdrl:HRAS-mCherry)^s916^* [14], which shows a membrane-localised endothelial expression and high CNR levels [13]. In this work, the same motion correction approach was investigated in the transgenic *Tg(fli1a:eGFP)^y1^* [15], which shows a pan-endothelial expression pattern and lower CNR levels than the former, thus evaluating the applicability of the suggested motion correction approach to more challenging data.

Enhancement of the vasculature in zebrafish was previously conducted using general filtering methods (Median filter and Rolling Ball algorithm) [13], providing increased vascular CNR levels.

However, our method’s performance was found to be sub-optimal when enhancing dim vessels or vessels with a strong cross-vessel double-peak intensity distribution. Hence, in this study, we present an alternative enhancement method utilizing the image Hessian matrix and the assumption of local vessel tubularity based on [16]. The cross-vessel intensity distribution response is assessed upon application of the newly suggested enhancement approach.

This work also evaluates several readily implemented segmentation methods available in the Fiji image analysis framework [17] and studies their applicability to segment the zebrafish vasculature after vascular enhancement.

Lastly, we quantify the cranial dorsal vascular volume and use this to provide an insight into the robustness of the vascular enhancement and segmentation methods implemented in this work.

## 2. Materials and Methods

### 2.1. Zebrafish Husbandry

All experiments were conducted in accordance with institutional and UK Home Office regulations under the project licence 70/8588 held by TC. Maintenance of adult transgenic zebrafish at the Bateson Centre Aquarium Facility at the University of Sheffield was conducted according to previously described standard husbandry protocols [18], with embryonic staging according to Kimmel et al. [19]. The following transgenic lines were studied:

1. *Tg(kdrl:HRAS-mCherry)^s916^* [14]: membrane tagged mCherry under the endothelial-specific kdrl promotor;
2. *Tg(fli1a:eGFP)^y1^* [15]: cytosolic eGFP under the pan-endothelial fli1a promotor;
3. *Tg(fli1a:LifeAct-mClover)^sh467^* (will be described elsewhere): endothelial filamentous actin tagged with mClover fluorophore under the pan-endothelial fli1a promotor.

### 2.2. Image Acquisition

Data acquisition in the whole dorsal cranial vasculature was performed using a Zeiss Z.1 light sheet microscope, Plan-Apochromat 20x / 1.0 Corr nd = 1.38 objective, dual-side illumination with online fusion and activated Pivot Scan at 28 Images were obtained with 16bit image depth, 0.334 µm × 0.334 µm × 0.69 µm voxel size, resolution 1920 × 1920 vx (x,y), and user-defined z-stack depth (typically 400–600 slices). Embedding of anaesthetized samples was conducted using 2%-LM agarose (Sigma-Aldrich, Dorset, UK) in E3 (5 mM NaCl, 0.17 mM KCl, 0.33 mM CaCl and 0.33 mM MgSO_4_ diluted to 1X E3 with distilled H_2_O) with 0.01% tricaine (MS-222, Sigma-Aldrich, Dorset, UK).

The following datasets were acquired:

1. Images of 3 days post fertilization (dpf) *Tg(fli1a:eGFP)^y1^*, *Tg(kdrl:HRAS-mCherry)^s916^*, and *Tg(fli1a:Lifeact-mClover)^sh467^* for CNR quantification;
2. Images of 2-to-5 dpf *Tg(kdrl:HRAS-mCherry)^s916^* for assessment of CNR, vascular enhancement, segmentation, and segmentation robustness;
3. Time-lapse acquisitions with 200 cycles over 10 minutes (min) were performed with 3 s time intervals in 3 dpf *Tg(kdrl:HRAS-mCherry)^s916^* and *Tg(fli1a:eGFP)^y1^* to assess extent of motion and test the motion correction approach.

### 2.3. Data Analysis

All image analysis, pre-processing, and segmentation were performed using the open-source software Fiji [17].

#### 2.3.1. Contrast-to-Noise Ratio (CNR)

Data quality and variability across a range of vessel sizes was assessed using CNR measurements. CNR was measured in the middle mesencephalic central artery (MMCtA), anterior cerebral vein (ACeV), basal aorta (BA) as well as dorsal aorta (DA), as these were found the be most consistent in location between samples, but showed a varying diameter. Regions of interest (spanning the whole vascular cross-section over a 5 µm length in the anterior-posterior direction) were placed in vessels of interest to measure vascular mean intensity (*µ_v_*). In the MMCtA and ACeV, the ROI was placed in the anterior-posterior middle of the vessel length. In the BA, the ROI was placed before its bifurcation into posterior communicating segment (PCS), while the DA ROI was placed at the anterior-posterior height of first intersomitic vessel (ISV). Non-vascular mean signal (*µ_nv_*) was measured in central regions of brain, without vascularization, at the same stack depth as the respective vessels to assess non-vascular signal. An estimate of background noise was obtained by measuring the standard deviation (*σ*) in a region of interest placed outside of the fish. CNR (Equation (1)) was calculated, as below:

(1) $$\mathrm{CNR}=\frac{\mu_{v}-\mu_{nv}}{\sigma }=\frac{\mathrm{mean}\;\mathrm{signal}\;-\;\mathrm{mean}\;\mathrm{non–vascular}\;\mathrm{signal}}{\mathrm{standard}\;\mathrm{deviation}\;\mathrm{of}\;\mathrm{background}}$$

#### 2.3.2. Image Pre-Processing 

Sample motion artefact assessment and correction were conducted as described in [13].

Two approaches for vascular enhancement were evaluated. The first utilised an optimised general filtering approach for image artefact and noise reduction [13], while the second was based around using image gradient information for enhancing tubular structures. Both methods were performed on data from the *Tg(kdrl:HRAS-mCherry)^s916^* line after 3D slice-by-slice motion correction as follows:

1. General Filtering (GF): 2D median filter with a radius of 6 voxels (13-by-13 neighbourhood) to remove local noise peaks and valleys [20] and a rolling ball algorithm of size 200 to suppress larger-scale artefacts such as scattering, autofluorescence, or shadowing artefacts [21]
2. Tubular Filtering (TF): Fiji Tubeness Plugin, based on Sato [16], and implemented by Mark Longair, Stephan Preibisch and Johannes Schindelin [17]. The effect of varying the TF scale parameter (sigma) on the vascular double-peak intensity distribution was evaluated for the following values: 5.3424 (16 px), 8.0232 (24 px), 9.3604 (28 px), 10.6848 (32 px), 15.359 (46 px), and 23.718 (69 px).

#### 2.3.3. Image Segmentation and Total Volume Measurement

Segmentation of enhanced images, generated using the two approaches described in the previous section, was performed to distinguish vascular from non-vascular information. Standard Fiji implementations of the following methods were evaluated [17]:

(i) Global Otsu thresholding using 16-bit images [22].
(ii) k-means clustering using 16-bit images [23], initialized using the default 48 randomized seeds automatically placed by the k-means++ algorithm [24]. Variation of seed number was not found to improve segmentation results in the tested range of 20–70 seeds. Additional parameters were set as follows: 0.0001 cluster centre tolerance and interpretation as 3D stack. Detection of four clusters was chosen as this was found to deliver reliable results, especially after TF (one background cluster and three vessel clusters with varying brightness).
(iii) Statistical region merging (SRM) using 8-bit images, due to input requirements of the Fiji implementation [25,26].
(iv) Level set [27] using 8-bit images (to achieve acceptable processing times) with 50 user-specified vascular seeds, using the “Fast Marching” option with a distance threshold of zero and user-selected image-specific grey value thresholds.

**Figure 1:**
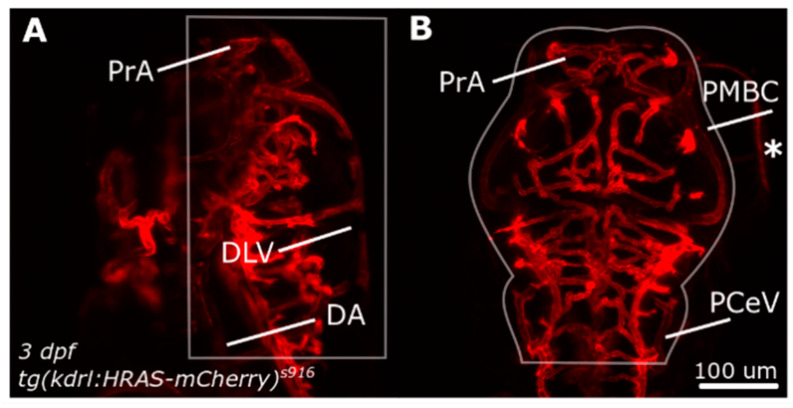
Dorsal cranial volume was measured in the region indicated by white outlines. (**A**) Dorsal-to-ventral boundary was established during image acquisition. (**B**) Lateral boundaries were defined by user to exclude structures outside the ROI, such as the eyes (asterisk). Figure reproduced with permission from [13] under licence 4466480468142.

Following segmentation, total dorsal cranial vascular volume (*Vol* (µm^3^), Equation (2)) was calculated in a user-defined region of interest (ROI; Figure 1). The dorsal aorta (DA) was chosen as the most ventral boundary, while the dorsal longitudinal vein (DLV) constituted the most dorsal during image acquisition. Exclusion of unspecified regions, such as the eye (indicated with asterix) were excluded via manual region of interest (ROI) selection. Anterior and posterior inclusion were based on prosencephalic artery (PrA) and posterior cerebral vein (PCeV), while lateral inclusion was guided by the anatomy of primordial midbrain channel (PMBC).

The total dorsal cranial vascular volume (*Vol* (µm^3^) was calculated by multiplying the total count of vascular voxels in the region of interest (*N_vasc_*, Figure 1) by the respective voxel volume (*V_x_*_,*y*,*z*_ (µm^3^)) as previously presented in [13].

*Vol* = *N_vasc_* ∗ *V*_*x*,*y*,*z*_ (2)

### 2.4. Statistics and Data Representation

In all cases, data conformity to a Gaussian distribution was tested using the D’Agostino-Pearson omnibus test to ascertain whether parametric statistical tests were appropriate [28]. Statistical analysis was performed using One-way ANOVA or paired Student’s t-test in GraphPad Prism Version 7 (GraphPad Software, La Jolla, CA, USA). The level of statistical significance was indicated by p-values using the following notation: *p* < 0.05 *, *p* < 0.01 **, *p* < 0.001 ***, *p* < 0.0001 ****. All graphical representations use mean values and standard deviation. Correlation analysis was performed using Pearson’s correlation coefficient. Image representation and visualization was done with Inkscape Version 0.48.

## 3. Results and Discussion

### 3.1. Image Pre-Processing

#### 3.1.1. Assessment of Image Quality by CNR Quantification

**Figure 2:**
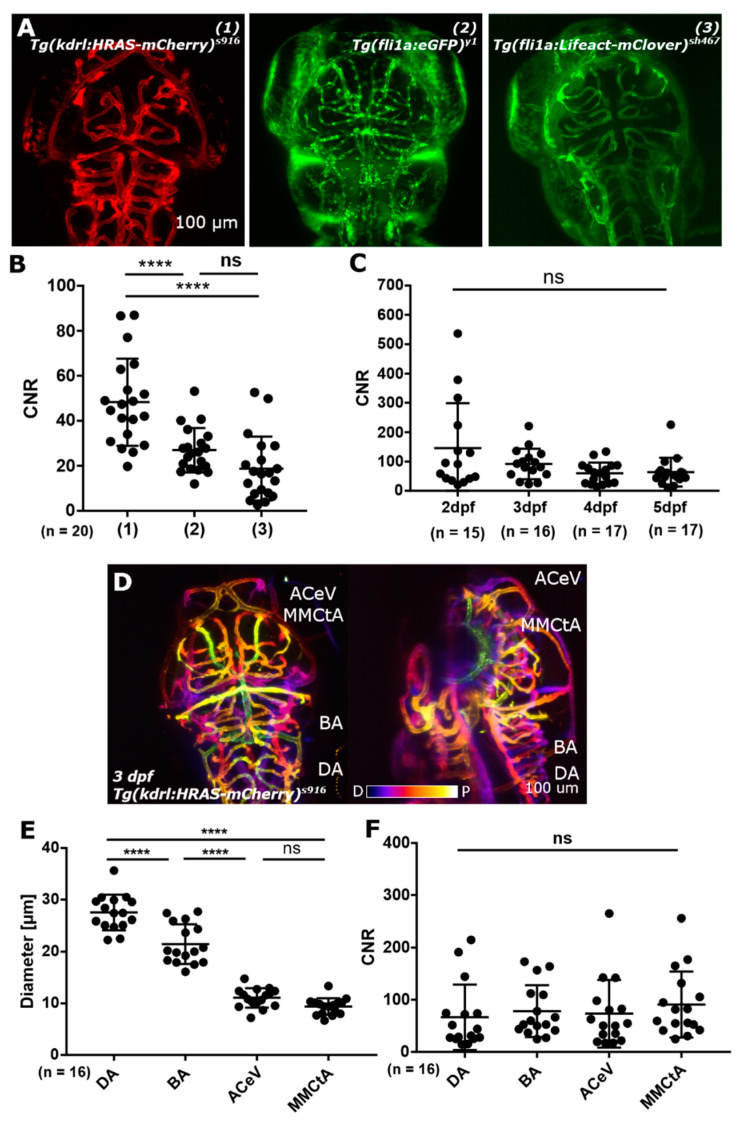
(**A**) Image quality was assessed by contrast-to-noise ratio (CNR) level measurement in three different transgenic lines, which harbour different reporter constructs (promotor as well as reporter; 1,2,3). (**B**) CNR levels in these transgenic reporter lines showed a statistically significant difference between (1) and (2) as well as between (1) and (3), with *p* < 0.0001 (****) for both. Between (2) and (3) no statistically significant difference was found (*p* 0.0898; ns). (**C**) CNR levels from 2-to-5 dpf showed no statistically significant difference (*p* 0.1032; ns). (**D**) Light sheet fluorescence microscopy (LSFM) allows to visualize vessels from the most dorsal plane (p - proximal image plane; e.g., anterior cerebral vein (ACeV)) to vessels which are a few hundred micrometer inside the embryo (d distal image plane; e.g., dorsal aorta (DA)). (**E**) Vessels in the dorsal cranial zebrafish vasculature with different diameters show no statistically significant difference in CNR levels ((**F**); *p* 0.3007 (ns); Figures (**A**) and (**D**) reproduced with permission from [13] under licence 4466480468142.).

After analysis of collated data from independent experimental repeats of CNR levels in the transgenics *Tg(kdrl:HRAS-mCherry)^s916^*, *Tg(fli1a:eGFP)^y1^*, and *Tg(fli1a:LifeAct-mClover)^sh467^* we, again found that *Tg(kdrl:HRAS-mCherry)^s916^* had the highest CNR level of the three tested transgenic lines (Figure 2A,B); being 48.26 ± 19.35 and statistically significantly higher than *Tg(fli1a:eGFP)^y1^* (26.92 ± 9.91) or *Tg(fli1a:LifeAct-mClover)^sh467^* (18.74 ± 14.20) (*p* < 0.0001 for both; n = 20, 4 dpf larvae; 3 experimental repeats). Differences between CNR levels in *Tg(fli1a:eGFP)^y1^* and *Tg(fli1a:LifeAct-mClover)^sh467^* were not statistically significant (*p* 0.0898), although there was a trend towards higher CNR in the former. The additional data obtained in this study did not change the overall trends of previously suggested image quality levels of the investigated transgenic reporter lines, but it did allow the CNR difference between *Tg(kdrl:HRAS-mCherry)^s916^* and *Tg(fli1a:eGFP)^y1^* to reach statistical significance.

Quantification of CNR in the basal artery (BA) from 2–5 dpf in *Tg(kdrl:HRAS-mCherry)^s916^* showed a slight but not statistically significant decrease during this developmental time-frame (Figure 2C; *p* 0.1032; 2 dpf n = 15, 3 dpf n = 16, 4 dpf n = 17, and 5 dpf n = 17 larvae; 2 experimental repeats; Kruskal-Wallis test); suggesting that vascular segmentation should not directly be influenced by CNR within this developmental time-frame.

Measurements of CNR levels in *Tg(kdrl:HRAS-mCherry)^s916^*, taken from vessels innervating the brain at different anatomical planes (Figure 2D) and with different diameters, showed that differences between anatomical location and vascular diameter (Figure 2E) were not correlated with differences in CNR levels (Figure 2F; *p* 0.3007; n = 16, 3 dpf larvae from 2 experimental repeats; average diameter DA = 22.28 ± 3.89 µm; basal artery (BA) = 11.14 ± 1.68 µm; anterior cerebral vein (ACeV) = 9.78 ± 2.10 µm; middle mesencephalic central artery (MMCtA) = 8.15 ± 1.27 µm; ANOVA; average CNR DA = 145.6 ± 153.1 (coefficient of variation (CoV) 105.11%); BA = 91.76 ± 52.18 (CoV 56.87%); ACeV = 59.55 ± 36.24 (CoV 60.86%); MMCtA = 63.91 ± 48.78 (CoV 76.33%); Kruskal Wallis). This suggests that segmentation of vessels of different size and anatomical location should not be influenced by varying CNR.

These data suggested that image segmentation was likely to be more challenging in the transgenic lines *Tg(fli1a:eGFP)^y1^* and *Tg(fli1a:LifeAct-mClover)^sh467^*, but not directly dependent on different CNR levels due to age (between 2-to-5 dpf), anatomical location, or diameter.

In this work, CNR was calculated using the mean signal across the entire vessel cross-section to provide an estimate of the signal within the vessel. However, as previously shown, the signal distribution is not uniform across the vessel cross section, so care must be taken in its interpretation, particularly if there is a strong edge response compared to the centre of the vessel. Nevertheless, CNR provides a simple quantitative measure that enables us to anticipate the likely feasibility of performing vascular segmentation in different transgenic lines [13]. 

#### 3.1.2. Correction of Motion Artefacts

**Figure 3:**
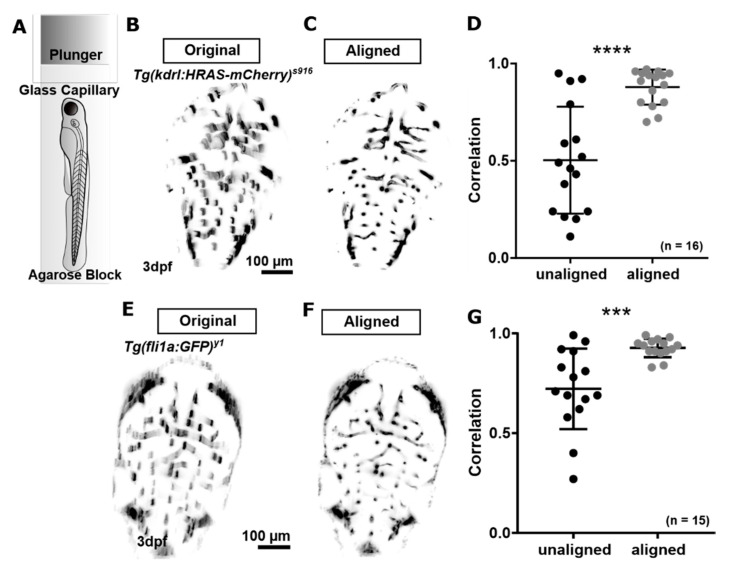
(**A**) Samples were embedded in an agarose block for LSFM. (**B**–**D**) Motion correction for the transgenic *Tg(kdrl:HRAS-mCherry)^s916^* was previously tested [13]. (**E**,**F**) Application of the same motion correction approach to the transgenic *Tg(fli1a:eGFP)^y1^* delivered a similar level of artefact correction (*p* 0.0008; ***). Figure (**D)** reproduced with permission from [13] under licence 4466480468142.

We previously investigated the extent to which motion artefacts occurred in our image acquisition setup using *Tg(kdrl:HRAS-mCherry)^s916^* (Figure 3B) and suggested a motion correction approach based on scale invariant features (Figure 3C,D; *p* < 0.0001; paired *t*-test; n = 16 3 dpf *Tg(kdrl:HRAS-mCherry)^s916^* larvae; 2 experimental repeats) [13,29]. Here, we applied the same principle of assessing and correcting for motion artefacts to the transgenic *Tg(fli1a:eGFP)^y1^* (Figure 3E). Application of motion correction significantly increased image correlation from 0.722 ± 0.202 to 0.927 ± 0.046 (*p* 0.0008; Figure 3G; paired *t*-test; n = 15 3 dpf larvae; 2 experimental repeats).

This suggested that the proposed motion correction method can also be applied to successfully correct for motion artefacts in other transgenic lines that exhibit significantly poorer image quality than *Tg(kdrl:HRAS-mCherry)^s916^*.

Based on our findings, we suggest that scale invariant features are suitable characteristics which can be used even in scarce data such as the vasculature. Future work needs to address intra-plane motion artefacts, which could be corrected by plane removal and interpolation from adjacent planes. Also, we appreciate that the extent of observed motion may differ for different sample embedding approaches such as fluorinated ethylene tubing [4] or different agarose percentage.

### 3.2. Vascular Enhancement and Segmentation

#### 3.2.1. Vascular Enhancement

**Figure 4:**
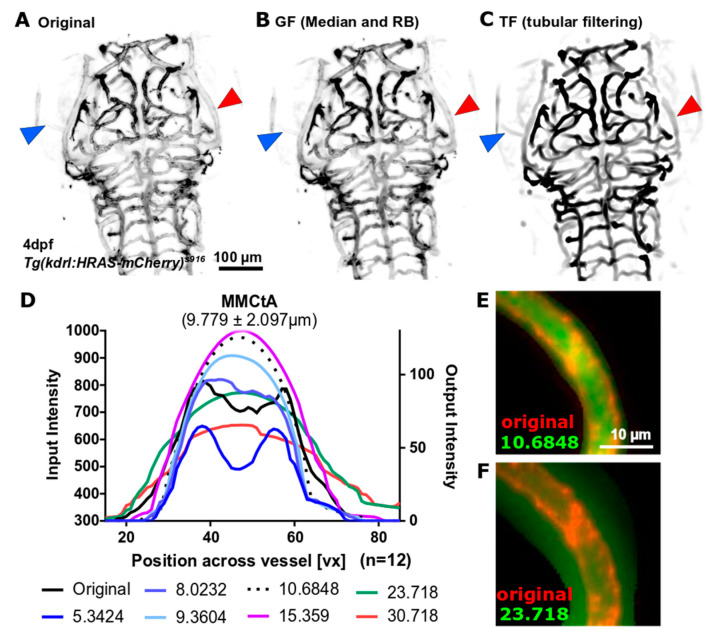
(**A**) Original data sometimes suffered from dimly visualized vessels (blue arrowhead) and had a double-peak cross-vessel intensity distribution (red arrowhead), which was particularly pronounced in bigger vessels. (**B**) Application of General Filtering (GF) failed to enhance dim vessels. (**C**) Application of Tubular Filtering (TF) successfully enhanced dim vessels and extracted vessels with double-peak intensity distributions as one segment (red arrowhead). (**D**) Assessment of the cross-vessel intensity distribution showed that the original double-peak distribution (black line; averages of n = 12 embryos) was successfully converted into a single-peak when scale reached vessel size (e.g., 9.3604 or 10.6848), while larger scales were found to reduce central intensity and blur vessel edges (e.g., 15.359 and above). This was further validated by visual investigation (**E**,**F**) where the TF response is shown in green overlaid onto the original image in red.

While application of GF generally resulted in poor enhancement of dimmer vessels and failed to enhance vessel double-peak intensity distribution as part of one vessel (Figure 4B; blue and red arrowhead, respectively), TF was found to overcome both these limitations and, moreover, delivered a visually overall better enhancement (Figure 4C). Assessment of cross-sectional intensity distribution after TF with different scale size (sigma), showed that the original double-peak intensity distribution was converted to single-peak when the maximum scale size reached approximately the size of the vessel (Figure 4D). Setting maximum scale beyond vessel diameter was found to reduce the central intensity peak and blur vascular edges far beyond original vessel edges (Figure 4E,F), highlighting the necessity for appropriate parameter selection. In the following the maximum scale was set to 10.5 because most cranial vessels were found to be in this size range (majority 7–13 µm; maximum 20 µm), suggesting that most vessels should be equally well enhanced, while blurring was avoided. Future work could include a combination of multiple scale responses to account for differences in vessel sizes and ensure an optimum response at every scale. 

#### 3.2.2. Segmentation Approaches

**Figure 5:**
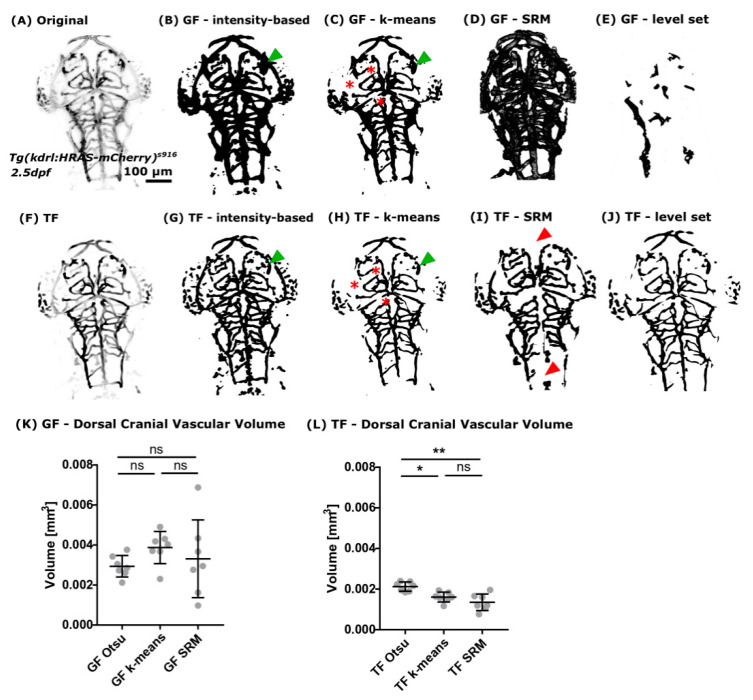
(**A**) Readily implemented segmentation methods of the Fiji image analysis software were applied to images after pre-processing using GF. (**B**) It was found that intensity-based Otsu-thresholding delivered the most robust results in comparison to other methods, such as k-means clustering ((**C**); red asterisks: vessels not extracted), SRM (**D**), or fast-marching level set (**E**). (**F**) The same segmentation methods were applied to images after vessel enhancement with TF. Again, it was found that intensity-based Otsu-thresholding delivered the most reliable results (**G**). While k-means clustering (**H**), SRM (**I**), and the tested fast-marching level set implementation (**J**) resulted in an unsatisfactory segmentation. (**K**) Vascular volume quantification after GF showed no statistically significant difference between the tested segmentation methods. (**L**) Quantification of the vascular volume after TF showed a statistically significant difference between the assessed segmentation methods. Namely, TF Otsu vs. TF k-means *p* 0.0293, TF Otsu vs. TF SRM 0.0058, and TF k-means vs. TF SRM *p* > 0.9999. CoV was found to be lower after TF with 15.24% k-means and 30.00% SRM and only 10.71% Otsu-thresholding (n = 7, Mann-Whitney U test; representative images).

We found that Otsu-thresholding delivered the most accurate segmentation after GF (Figure 5B) as well as TF (Figure 5G). Subjectively comparing GF and TF, it was found that extracted vascular features after GF sometimes showed increased blurring and/or less detail (Figure 5, green arrowheads).

Assessing the k-means clustering segmentation output showed that dim vessels were not well extracted and non-vascular regions adjacent to vessels were over-segmented, leading to over-segmentation of vessel width (Figure 5C,H, red asterisk).

The segmented output after SRM was often under- or over-segmented (Figure 5D,I) and loss of whole vascular segments was occasionally observed (Figure 5I, red arrowheads). Again, vessel width was found to be over-segmented, leading to highly variable results after GF. The low variation of SRM after TF is thought to be due to suppression of non-vascular regions during the enhancement process.

Application of the fast-marching level set method to our data resulted in unsatisfactory segmentation outputs with vessels generally being under-segmented when compared to the other approaches. This was particularly noticeable when the method was combined with the GF enhancement approach. Also, due to data size, computational time was approximately 2–3 days on a PC workstation, suggesting that this method would not be feasible in this current implementation for large datasets without prior image down-sampling. It also precluded any detailed attempt to optimize the methodology, as evaluating the method over a range of input parameters was not considered feasible in the time-frame of the present study. Thus, level set methods are not considered further in the present study.

Quantification of the cranial vascular volume after segmentation was performed to evaluate robustness and replicability of the suggested segmentation approach. Comparing the extracted vascular volume after GF enhancement showed no statistically significant difference for any of the three tested methods, Otsu-thresholding, k-means, and SRM, respectively (Figure 5K; GF Otsu vs. GF k-means *p* 0.1742; GF Otsu vs. GF SRM *p* > 0.9999; GF k-means vs. GF SRM *p* 0.5454). Interestingly, the CoV varied strongly between the suggested methods after GF, namely Otsu-thresholding 18.34%, k-means 20.79%, and SRM 58.59%. Suggesting that Otsu-thresholding delivered the most consistent segmentation.

Quantification of the vascular volume after TF enhancement showed statistically significant differences between the suggested segmentation methods as follows: TF Otsu vs. TF k-means, *p* 0.0293; TF Otsu vs. TF SRM, *p* 0.0058; and TF k-means vs. TF SRM, *p* > 0.9999 (Figure 5L). Again, Otsu-thresholding provided the lowest CoV of 10.71%, while the CoV for k-means and SRM were 15.24% and 30.00%, respectively.

Based on the high CoV after GF we suggest that TF enhancement prior to segmentation delivered more robust and reliable results in comparison to GF. Also, our data imply that Otsu-thresholding delivered more accurate segmentation results than k-means or SRM. Together, we suggest that enhancement with TF followed by Otsu-thresholding can be used to extract useful quantitative measures of the cranial vasculature volume.

This vascular enhancement and segmentation approach has been optimized and validated using the reporter line *Tg(kdrl:HRAS-mCherry)^s916^* which was shown in Section 3.1.1 to have high image quality and endothelial specific expression. Hence, future studies will need to investigate the translation of the approach to other transgenic reporter lines with lower CNR levels and/or more unspecific fluorophore expression. Furthermore, while we have demonstrated that the optimum segmentation approach provides the most precise measurement of vascular volume, the current lack of a gold standard makes it difficult to assess the accuracy of the method and this work has relied on subjective visual assessment to provide a gauge, which is far from ideal. Future work will need to address the lack of a gold standard for the segmentation of the zebrafish vasculature. Because no technique is currently available as the gold-standard for zebrafish vascular segmentation, laborious manual segmentation would be required. Instead, we suggest further investigation of accuracy and sensitivity by the use of experimental approaches (such as inhibition of angiogenesis to assess segmentation sensitivity), computational approaches (such as vasculature modelling to refine following parameter settings), or both in combination.

## 4. Conclusions

In this work, we have investigated image pre-processing and segmentation approaches to provide a work-flow for quantitatively characterising the zebrafish cranial vascular volume. We have shown that inter-plane motion correction can be successfully corrected utilizing scale invariant features, as demonstrated in two different transgenic lines, which have different data properties. We have also shown that CNR varies between different transgenic reporter lines, but is largely independent of age (between 2-to-5 dpf), anatomical location, or vessel size. Testing GF and TF for vascular enhancement, we found that TF delivered optimum results when the scale size was in the range of “to be enhanced” vessels. Following, we tested different segmentation methods and found that the combination of TF and Otsu thresholding allowed for robust quantification of the cranial vascular volume. Thus, we suggest that biologically relevant information can be extracted to study vascular development, disease influence, or effect of drugs. This segmentation of the cranial vasculature will be the foundation for future extraction of further physiologically relevant data, such as vessel length, branching point, or diameter.

Future work may also consider the collation of vessel enhancement data from multiple scales to allow application to vascular beds with a wider range of vessel sizes. Also, being able to visualize vessel walls of lumenised vessels by the use of transgenic lines and LSFM could allow future approaches to quantify vessel wall thickness and luminal volumes for the further understanding of cardiovascular diseases.

## Abbreviations

The following abbreviations are used in this manuscript:

ACeV	anterior cerebral vein
BA	basal artery
CNR	contrast-to-noise ratio
CoV	coefficient of variance
DA	dorsal aorta
DLAV	dorsal longitudinal vein
dpf	days post fertilization
GF	general filtering
hpf	hours post fertilization
H	Hours
ISV	intersomitic vessel
LP	laser power
LSFM	light sheet fluorescence microscopy
min	Minutes
MIP	Maximum intensity projection
MMCtA	middle mesencephalic central artery
PCeV	posterior cerebral vein
PCS	posterior communicating segment
PMBC	primordial midbrain channel
PrA	prosencephalic artery
ROI	region of interest
s	Seconds
SRM	statistical region merging
TF	tubular filtering
